# Quantification of Pseudouridine Levels in Cellular RNA Pools with a Modified HPLC-UV Assay

**DOI:** 10.3390/genes8090219

**Published:** 2017-09-05

**Authors:** Jialin Xu, Alice Y. Gu, Naresh R. Thumati, Judy M.Y. Wong

**Affiliations:** Faculty of Pharmaceutical Sciences, University of British Columbia, Vancouver, BC V6T 1Z3, Canada; jialin.xu@alumni.ubc.ca (J.X.); alicebgu@hotmail.com (A.Y.G.); nareshthumati@gmail.com (N.R.T.)

**Keywords:** pseudouridine, dyskerin, HPLC, RNA modification

## Abstract

Pseudouridine (Ψ) is the most abundant post-transcriptionally modified ribonucleoside. Different Ψ modifications correlate with stress responses and are postulated to coordinate the distinct biological responses to a diverse panel of cellular stresses. With the help of different guide RNAs, the dyskerin complex pseudouridylates ribosomal RNA, small nuclear RNA and selective messenger RNAs. To monitor Ψ levels quantitatively, a previously reported high performance liquid chromatography method coupled with ultraviolet detection (HPLC-UV) was modified to determine total Ψ levels in different cellular RNA fractions. Our method was validated to be accurate and precise within the linear range of 0.06–15.36 pmol/μL and to have absolute Ψ quantification levels as low as 3.07 pmol. Using our optimized HPLC assay, we found that 1.20% and 1.94% of all ribonucleosides in nuclear-enriched RNA and small non-coding RNA pools from the HEK293 cell line, and 1.77% and 0.98% of ribonucleosides in 18S and 28S rRNA isolated from the HeLa cell line, were pseudouridylated. Upon knockdown of dyskerin expression, a consistent and significant reduction in total Ψ levels in nuclear-enriched RNA pools was observed. Our assay provides a fast and accurate quantification method to measure changes in Ψ levels of different RNA pools without sample derivatization.

## 1. Introduction

Of the 166 known RNA nucleoside modifications, pseudouridine (Ψ) is the most abundant post-transcriptional modification and was the first to be discovered [1,2]. The isomerization of 1-ribosyluracil (uridine) to 5-ribosyluracil provides an additional hydrogen bond donor on the uracil base that contributes to the stabilization of base-stacking and RNA structures [3].

Known as the fifth ribonucleoside, Ψ is widely distributed in almost all RNA-containing species and constitutes over 1% of total nucleosides and over 7% of all uridines in ribosomal RNA (rRNA) fractions. Ψ has been shown to enhance transfer RNA (tRNA) binding to ribosomes and translational fidelity of rRNAs [4,5]. It is also involved in pre-mRNA splicing regulation in spliceosomal small nuclear RNAs (snRNA) and in decoding and stabilizing tRNA (reviewed in [6]).

Recently, with the advent of massive parallel sequencing of the transcriptome, hundreds of naturally modified Ψ sites in mRNA were discovered in yeast and human cells [7,8,9]. Specifically, around 0.2–0.6% of uridines in mammalian mRNA are pseudouridylated [10]. Pseudouridylation profile in mRNA was dynamically regulated in a stress-specific manner [7,8,10]. Although the biological consequences of mRNA pseudouridylation remain unknown, it is postulated that the irreversible changes in the pseudouridylation profile of the transcriptome could result in functional changes including protein recoding, reduced translational efficiency, and altered transcript structure [11].

In humans, there are 13 known Ψ synthases (PUSs) that can catalyze the isomerization of uridine to Ψ [12]. Based on their respective mechanisms of action, these enzymes can be classified into RNA-independent and RNA-dependent PUS families. RNA-independent PUS enzymes promote Ψ formation with classical enzymatic catalysis, through protein–RNA interactions between the enzyme’s catalytic domain and the structural context of the modified target uridine. In contrast, Ψ modifications of specific rRNA and snRNA residues are carried out by the box H/ACA ribonucleoprotein (RNP) complex in a guide RNA-dependent manner.

The H/ACA RNP complex is comprised of one box H/ACA small nucleolar/Cajal body RNA (snoRNA or scaRNA) and two copies of a protein catalytic subunit comprised of dyskerin, GAR1, NHP2, and NOP10. The catalytic core, formed by the dyskerin/NHP2/NOP10 trimers complexed with a single guide RNA, was assembled through sequential binding with biogenesis factors including SHQ1 and NAF1 [13]. GAR1 brings the mature H/ACA RNP assembly to Cajal bodies and nucleoli, where nascent target RNAs are modified [14]. The specificity of pseudouridylation in this system comes from base-pair interactions between the antisense elements in the box H/ACA snoRNA and the sequence of the target RNA. Guided by the snoRNA, the dyskerin/NHP2/NOP10 complex carries out pseudouridylation of specific uridine residues in non-coding, as well as coding RNA [3].

While the dyskerin/NHP2/NOP10 complex is the only known RNA-dependent PUS, it is responsible for the modification of various targets by virtue of its ability to switch RNA guides. All box H/ACA guide RNAs are non-coding small RNAs with a hairpin–hinge–hairpin structure and the signature ACA residues at the 3′ terminus at maturation [15]. The most abundant H/ACA-RNAs are found localized at the nucleolus (H/ACA-snoRNAs). These guide RNAs are responsible for directing the majority of rRNA Ψ modifications. In comparison, H/ACA-scaRNAs have much greater structural diversity than H/ACA-snoRNAs, with a subset of scaRNAs containing both guiding Ψ (H/ACA) and guiding 2′-*O*-methyl (structural C/D box motif) modifications. H/ACA-scaRNAs are found localized to the Cajal body and are involved in the nucleoside modifications of selected small nuclear RNAs involved in the spliceosome complex [16].

Since Ψ’s discovery, multiple methods have been developed to map the location and quantify the levels of Ψ. The most popular method for studying Ψ in RNA is based on chemical derivatization of RNA by *N*-cyclohexyl-*N*′-β-(4-methylmorpholinium)ethylcarbodiimide (CMC) *p*-tosylate [17]. The CMC *p*-tosylate reacts with all guanosine, uridine, guanosine-like, and uridine-like residues to form adducts. A subsequent cleavage step hydrolyzes all adducts except those formed at the Ψ bases [5,18]. As the bulky CMC group at the Ψ site sterically stops reverse transcription, truncated products from the primer extension assay with reverse transcriptase will be able to sequence-specifically determine the location of each Ψ modification site. Although this method is useful for the determination of site-specific pseudouridylation, the derivatization, cleavage, and reverse transcription steps introduce variables that may influence accurate quantification.

In contrast, the traditional physicochemical property-based chromatography method provides an alternative way to quantify relative Ψ levels by simple enzymatic digestion of cellular RNA into single nucleosides [19]. Upon separation of different nucleosides by high-performance liquid chromatography (HPLC), different detection methods have been applied to determine Ψ levels in cellular RNA pools. Absorbance detectors, such as ultraviolet (UV) detectors measuring the light absorbing capacity of the analytes, are most commonly used for HPLC analysis. Previously, Tomikawa et al. established an HPLC-UV method for the detection and determination of ten different modified RNA nucleosides from the same digested RNA sample in a single injection [20]. However, this method was used semi-quantitatively for the comparison of relative RNA modification levels among different bacterial strains. In addition, the chromatographic conditions required an extensive 85 min for the analysis of one sample, making this method less desirable as an efficient screening assay.

Following liquid chromatography, mass spectrometry (MS) is also a popular detection choice for Ψ level measurements in recent years [21]. The fragmentation of molecules by electric fields provides individual molecules different base-to-charge ratios. Triple quadrupole MS was used to quantify modified nucleosides in a 40 min HPLC-MS run [22]. The improved sensitivity and specificity of newer generation MS facilitated the quantification of over 20 different modified nucleosides simultaneously. Efficiency of nucleoside analysis was also boosted by substituting HPLC with ultra performance liquid chromatography (UPLC), where a single sample could be analyzed within 15 min [23]. Sensitivity of nucleoside quantification is vastly improved with these newer methods using coupled LC/MS. However, such comprehensive profiling of RNA modifications may not be necessary for many simple research questions, considering the high cost of MS analysis and the availability of facilities.

In the current study, a previously reported HPLC-UV method [24] was modified to determine the Ψ levels in various cellular RNA pools. The modified method was successful in detecting changes in Ψ levels upon knockdown of dyskerin expression. Our measurement of Ψ levels remained precise and accurate at picomole levels. We contend that the modified HPLC-UV assay provides an economic option for rapid screening of small changes in Ψ level.

## 2. Methods

### 2.1. Chemicals and Expression Vector

The chemical reference compounds Ψ and 7-methylguanosine (7-metG) were obtained from Santa Cruz Biotechnology (Santa Cruz, CA, USA) and Sigma-Aldrich (St. Louis, MO, USA), respectively. Nucleoside test mix from Sigma was used as a reference to identify major nucleosides (A, U, G, C) in RNA pools. The aqueous mobile phase was prepared with ammonium dihydrophosphate from Sigma-Aldrich (St. Louis, MO, USA). Methanol (HPLC grade) was obtained from Fisher Scientific (Pittsburgh, PA, USA). Ultrapure water was obtained from a MilliQ water purification system (Millipore, Bedford, MA, USA). A previously described small hairpin (sh) RNA construct was applied to reduce dyskerin expression level by RNA interference [25].

### 2.2. Cell Culture and Transfection 

The human embryonic kidney cell line HEK 293 was obtained from American Type Culture Collection (ATCC, Manassas, VA, USA). The human cervical adenocarcinoma cell line HeLa was a generous gift from Dr. Eric Jan’s laboratory. Cells were cultured in 1X DMEM high glucose media (Gibco-BRL, Grand Island, NY, USA) supplemented with 5% fetal bovine serum and maintained at 37 °C with 5% CO_2_. Cells were trypsinized, quantified using a Coulter counter (Beckman Coulter Inc., Hialeah, FL, USA), and seeded at a density of 3 million cells per 100 mm plate. Cells were allowed to recover overnight until they were 40–60% confluent at the time of transfection.

Standard calcium phosphate mediated transfection was performed. The cells were maintained under puromycin selection to inhibit the growth of cells that did not express the shRNA plasmid. Cells were harvested 96, 120, and 144 h after the transfection.

### 2.3. RNA Extraction and Digestion

For collection of nuclear-enriched RNA pool, cells were resuspended in hypotonic lysis buffer (20 mM HEPES pH 8.0, 2 mM MgCl_2_, 0.2 mM EGTA, 10% glycerol, 1 mM DTT, 0.1 mM PMSF) and subjected to four consecutive freeze–thaw cycles in liquid nitrogen and in a 37 °C water bath. Following centrifugation at 1000× *g* for 15 min, the supernatant containing hypotonic lysis buffer and cytoplasmic components was removed. The remaining pellet was washed once with hypotonic lysis buffer. The nuclear RNA pool was then extracted with Trizol (Invitrogen, Carlsbad, CA, USA).

Total RNA was extracted with Trizol. Total RNA was separated by size on denaturing urea-polyacrylamide (PAGE) gel electrophoresis and the gel fraction between the region with xylene cyanol dye migration (~75 nt) and 5S rRNA (121 nt) was harvested. The excised band was sliced into small pieces with a clean razor and moved to a 15 mL conical tube. To the sliced gel pieces, 3× vol. of 0.3 M NaOAc (pH 5.2) and 0.1× vol. of phenol-chloroform were added. The tube was kept shaken at 37 °C overnight. Nucleic acid in the supernatant was extracted and precipitated with ethanol. Similarly, 18S and 28S rRNA were separated by size using a denaturing formaldehyde agarose gel, gel purified by the Ultrafree-DA centrifugal filter unit (Millipore, Bedford, MA, USA), and precipitated with ethanol.

RNA levels were quantified using Nanodrop (Nanodrop Technologies, Wilmington, DE, USA), and the concentrations of selected RNA samples were verified with the Ribogreen assay (Thermo Fisher Scientific, Waltham, MA, USA). Five micrograms of each RNA sample were sequentially hydrolyzed by five units of RNase T2 (Worthington Biochem, Freehold, NJ, USA) with 2× RNase T2 buffer (100 mM NaOAc, pH 4.5, and 2 mM EDTA) and dephosphorylated by 5 units of Shrimp Alkaline Phosphatase (Invitrogen, Calsbad, CA, USA) with 10× SAP buffer (100 mM Tris·HCl pH 8.0, 100 mM MgCl_2_, and 1 mg/mL BSA) following two overnight incubations at 37 °C. The final volume of each digested RNA sample was adjusted to 200 μL and 210 pmol of 7-metG was added to each sample as an internal control.

### 2.4. Chromatographic Conditions

Nucleoside separation and quantification were performed with a Waters 2695 HPLC system and Waters 2996 UV detector (Waters Corp., Milford, MA, USA). Chromatographic separation was performed with a 4 μm Waters Nova-Pak C18 column 3.9 mm × 150 mm (Waters Corp., Milford, MA, USA). The column was kept at room temperature, and the detection wavelength was set at 254 nm. The two mobile phase components were adapted from a previous study [24]. Mobile Phase A (0.01 M ammonium dihydrophosphate adjusted with phosphoric acid to pH 5.1) was filtered through a 0.22 μm membrane filter (Millipore, Bedford, MA, USA) before use. Mobile Phase B was composed of methanol/water (1:1 v/v). A linear gradient was programmed from 0 to 40% Mobile Phase B over the first 30 min, followed by 40% to 0% in the next minute, and then re-equilibration with 100% Mobile Phase A for 4 min [24]. The flow rate was set at 1 mL/min, and the injection volume was 50 μL. Data acquired were processed with Empower software.

### 2.5. Method Validation

Stock solutions of Ψ were made by dissolving the appropriate amount of pure Ψ in water to yield a final concentration of 200 ng/μL. Working solutions with concentrations of 0.10, 0.26, 0.64, 1.6, 4, 10, and 25 ng/μL was prepared every month by a 2.5-fold serial dilution from the stock. Calibration curves for Ψ levels were created on each analysis day by diluting 30 μL of working solution with water and digestion enzyme buffers into 200 μL (the final concentration of Ψ is equivalent to 0.06, 0.16, 0.39, 0.98, 2.46, 6.14, and 15.36 pmol/μL). The analyte to internal standard (I.S.) peak area ratios were plotted against matched amounts of Ψ added in the injected blank sample. The calibration curves were calculated by the least squares method. Linearity was assessed by determining the coefficient of correlation (*r*^2^) of data points on the plotted curves. The absolute Ψ amount was expressed as picomoles per microliter (pmol/μL) and converted to picomoles later.

For method validation, spiked samples were prepared at three different concentrations that cover the low, middle, and high ranges of the standard curve. Relative error (RE) between the nominal and measured concentrations was expressed as accuracy of the method, whereas relative standard deviation (RSD) of repeated measurements was expressed as precision.

Sensitivity of the method was estimated using the calibration curve method. The limit of detection (LOD) and limit of quantitation (LOQ) of the present method were calculated by the following formula: *A* = *kσ*/*S*, where *A* was LOD or LOQ, *k* was the coefficient for the two parameters (*k* = 3.3 for LOD and *k* = 10 for LOQ), *σ* was the standard deviation of the response (i.e., the intercept of the calibration curve), and *S* was the slope of the curve.

Specificity of the nucleosides in RNA pools was tested by injecting a nucleoside test mix and referring to the relative retention time in the standard.

### 2.6. Protein Expression Measurement by Western Blot

The Western blot protocol was the same as described in [26]. Whole cell extracts were quantified by Bradford protein assay and 30 μg of protein was resolved in 10% SDS-PAGE gel. After transfer of the resolved protein samples to a PVDF membrane, the blots were incubated with anti-dyskerin rabbit polyclonal antibodies (200 ng/mL, 1:1000 dilution, Santa Cruz Biotech, Santa Cruz, CA, USA) and anti-β-actin mouse monoclonal antibody (50 ng/mL, 140,000 dilution, Sigma-Aldrich, St. Louis, MO, USA). Alexa Fluor 680 goat anti-rabbit IgG and Alexa Fluor 790 donkey anti-mouse IgG were used as secondary antibodies (both from Thermo Fisher Scientific, Waltham, MA, USA, 1:10,000 dilution). The signals were detected by Licor Odyssey CLx Infrared Imaging System (LI-COR Biosciences, Lincoln, NE, USA) and analyzed by ImageJ software [27].

### 2.7. Statistical Analysis

Data were analyzed using GraphPad Prism software (GraphPad Software, San Diego, CA, USA). Error bars denote SEM. The one-way ANOVA with post-hoc Bonferroni correction was applied to adjust for multiple comparisons. Differences were considered significant at *p* < 0.05.

## 3. Results

### 3.1. Method Validation

**Figure 1:**
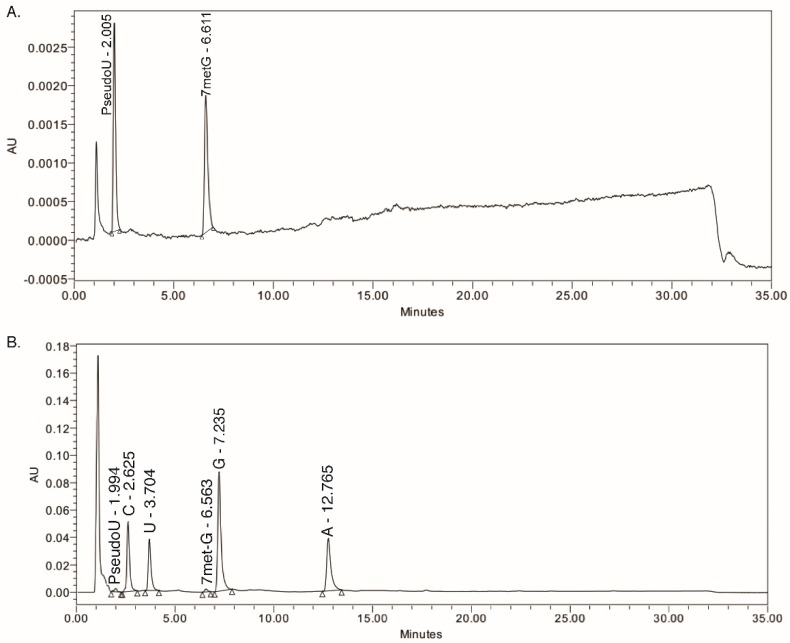
Representative high performance liquid chromatography (HPLC) chromatograms of (**A**) a pseudouridine (Ψ) quantification standard sample at 2.46 pmol/μL and (**B**) a digested nuclear-enriched RNA sample from the HEK293 cell line. AU: absorption units.

Representative chromatograms of a calibration standard sample and a digested RNA sample are shown in Figure 1A,B, respectively. The retention times for Ψ and 7-metG were 2.0 and 6.5 min. A solvent peak resulting from digestion buffers was identified in control experiments. Although other RNA nucleosides were not involved in our quantification method, their retention times were also stable and are listed in Table 1.

The peak area ratios of the calibration standard were proportional to the concentration of Ψ input over the range of 0.06–15.36 pmol/μL. The means ± SDs of four standard curve slopes and their intercepts for Ψ were 0.768 ± 0.059 and 0.002 ± 0.001, respectively. The regression coefficients (*r*^2^) of all calibration curves were greater than 0.999. (Representative calibration curves were shown in Supplementary Figure S1). Therefore, the LOD for this method was estimated to be 0.01 pmol/μL and LOQ estimated to be 0.02 pmol/μL.

Assay accuracy and precision were shown in Table 2. Since the above data showed that our method was valid for measuring a wide range of Ψ levels, we first determined the average Ψ levels in different cellular RNA pools and then applied this validated method to RNAi-mediated knockdown of dyskerin expression.

### 3.2. Average Ψ Levels in Different Cellular RNA Pools 

From the standard curve, the mean concentration of Ψ in nuclear-enriched RNA samples was found to be 0.88 pmol/μL. The total amount of Ψ involved in the injected sample (50 μL of RNA sample containing 1.25 μg of nuclear RNA) was calculated to be 44 pmol. Since 1.25 μg of the starting material, i.e., intact nuclear RNA is equal to 3.68 nmol, we concluded that approximately 1.20% of nucleosides in nuclear-enriched RNA are Ψs. This is in agreement with previous reports [3].

Using the same method, the amount of Ψ in 18S and 28S rRNA from HeLa cells in log-phase growth were calculated to be 1.77% and 0.98%, respectively (Table 3). It is known that in 28S rRNA (found in the 60S large ribosomal subunits), 57 out of 5025 nucleosides (1.13%) could be pseudouridylated [1]. Our result indicated that 86.7% of the maximum calculated level of Ψ in 28S rRNA in HeLa cells was indeed pseudouridylated. For 18S rRNA, 1.87% (35/1868) of total nucleosides are reported to be pseudouridylated [1], and our result of 1.77% was comparable to this value (94.7% of the maximum level). Thus, our experimental data agreed with published data on the extent of Ψ modifications within rRNAs.

It was also found in our study that 1.94% of gel-purified small RNA populations are pseudouridylated. The average Ψ concentration measured by our assay, and the equivalent amount and levels in different cellular RNA pools are listed in Table 3. While it is beyond the scope of our current report, we have previously shown that our Ψ quantification method can expand to include the measurement of Ψ levels in RNAs isolated through purification of specific RNP complexes, such as the 40S and 60S ribosomal subunits [28], thereby restricting Ψ analysis in functionally distinct populations of mature rRNAs. Similarly, we envisioned that Ψ quantifications of RNA components in specific RNP complexes could be analyzed following immunoprecipitation-purification.

### 3.3. Ψ Reduction Is Not Proportional to Dyskerin Knockdown

Next, we applied our assay to quantify changes in Ψ levels upon the reduction of dyskerin expression. Only 3 out of 13 known PUSs can catalyze pseudouridylation in rRNAs [12]. Out of the three enzymes, the dyskerin complex is the only mechanism that uses guide RNAs to target the positions of rRNA pseudouridylation. At the time of our work, the dyskerin complex was known to modify 35 sites on 18S rRNA, 57 sites on 28S rRNA, and 2 sites on 5.8S rRNA comprising the major rRNA Ψ modification mechanism [1,12,29,30].

We reasoned that as rRNA are known to have long half-lives (varying from 3 to 8 days in mammalian cells [31,32,33]), stable rRNA pools in cytoplasm may not reflect the changes in Ψ modification rates upon dyskerin knockdown. To enrich for nascent rRNA, we isolated the nuclear-enriched cell fractions, where newly transcribed rRNAs are modified. We expected to see a proportional relationship between Ψ levels and dyskerin protein expression in the nuclear fractions.

**Figure 2:**
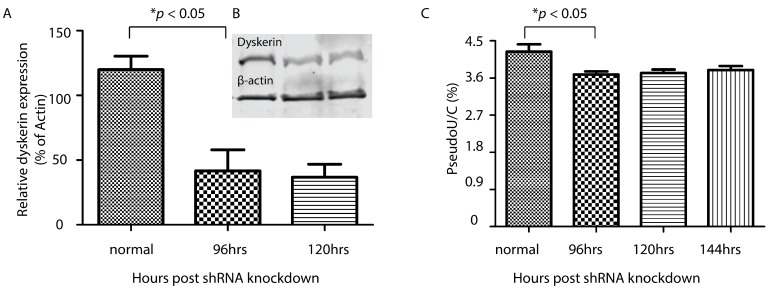
A reduction of Ψ was observed after dyskerin knockdown. (**A,B**) Representative quantification and immunoblot of dyskerin protein expression at 96 and 120 h after dyskerin knockdown with short hairpin RNA (shRNA); (**C**) Change in Ψ expression after dyskerin knockdown with shRNA. Ψ level was expressed as a function of cytosine (Ψ/**C**).

The HEK293 cells were directed to express a shRNA duplex against dyskerin and kept under antibiotic (puromycin) selection for at least 48 h. Antibiotic selection inhibited the growth of cells that were negative for the expression of the shRNA construct. With this approach, we confirmed that dyskerin expression in HEK293 cells was reduced to ~45% of regular levels (Figure 2A,B, *p* = 0.0188). As expected, there was also a consistent and significant reduction in Ψ levels in nuclear RNA fractions 96, 120, and 144 h post-shRNA transfection (Figure 2C, *p* = 0.0291). The reduction was not directly proportional to the reduction in dyskerin expression. Although HEK293 cells showed widespread cell death (data not shown), suggesting that they were under severe stress with the reduction in dyskerin expression, the average Ψ levels in these shRNA-treated cells were only ~10% lower than the control group (no dyskerin shRNA transfection).

## 4. Discussion

We developed a quantitative HPLC-UV-based assay to determine Ψ, the most abundant post-transcriptionally modified RNA base. We found that Ψ made up of 1.20% and 1.94% of all nucleosides in nuclear RNA and small RNA pools from the HEK293 cell line and 0.98% and 1.77% of nucleosides in 28S and 18S rRNA from the HeLa cervical cancer cell line. These results are comparable to previous reports [1,3].

Our linear range for quantification was 0.06–15.36 pmol/μL, which is equivalent to 3.07–767.81 pmol of Ψ in this assay. We calculated the mean amount of Ψ in 1.25 µg of nuclear RNA pools to be 44.16 pmol. With the assay being able to determine Ψ levels as low as at the picomole level, theoretically, the minimal amount of RNA input prior to the digestion step could be as low as ~100 ng (assuming the average Ψ content of the RNA sample being 1.20%, the same as the level in nuclear RNA pools in our assay). This is an easily collected sample amount from biochemical experiments, as well as from clinical/patient sources.

The LOD and LOQ for our assay were estimated to be 0.01 pmol/μL and 0.02 pmol/μL, respectively. These values equaled 0.31 and 0.92 pmol of Ψ or 10 and 31 ng of RNA input (assuming the percentage of pseudouridylation in the RNA sample for detection is around 1%). As well, the intra-day and inter-day coefficients of variation for precision and accuracy were below 10% for concentrations tested within the quantification range. In other words, minimal changes in Ψ levels as low as 0.31 pmol in RNA samples may be accurately and precisely analyzed by the current assay. Therefore, our assay provides a sufficient window for measuring changes in pseudouridylation under different conditions.

Based on the observed retention times, the major nucleosides were well separated. Importantly, Ψ had a stable retention time of 2.0 min that allowed it to be easily identified. Our method has already been applied to quantify the steady-state Ψ levels in rRNA samples from XDC patients with mutations in the dyskerin-encoding *DKC1* gene [26,28]. We found that specific mutations of the *DKC1* gene, but not all disease-associating mutations, led to reproducible but modest loss of steady-state Ψ levels in rRNA. We contended that, while these moderate changes in rRNA Ψ levels were not enough to drive the disease, they could nonetheless modulate the rate of disease onset and/or severity by a mechanism that involves reduced stress tolerance conferred by optimal rRNA Ψ modifications.

The observed non-proportional relationship between Ψ levels and dyskerin expression may be explained by the existence of different mechanisms for pseudouridylation. There are at least 12 additional PUSs known in humans, on top of the H/ACA RNP complex. Specifically, pseudouridylations in rRNA by PUS7 and PUS7L are carried out in a guide-RNA independent manner and are unaffected upon the knockdown of dyskerin expression [12]. On the other hand, *DKC1* is an essential gene in metazoan, and knockout of *DKC1* leads to embryonic lethality in mouse models [34]. It is thus conceivable that the reduction of dyskerin expression beyond a threshold level is cytotoxic. In agreement, we observed that, despite the use of puromycin selection over the course of 144 h, dyskerin protein expression did not reduce beyond 55%. This level of dyskerin expression may represent the minimal level compatible with life; thus, reduction of dyskerin expression to these levels may cause minimal disruptions to rRNA modification and the structure of the protein synthesis machinery.

It is worth nothing that the nuclear-enriched RNA pool we collected from our shRNA experiments is comprised of almost all organelles, including the nucleus, ribosomes, endoplasmic reticulum, Golgi body, cell membrane, and mitochondria. This RNA collection contains not only nascent rRNA, but also includes mRNA, tRNA, and other small RNAs. As dyskerin is not known to be substantially involved in the modifications of these other RNA populations, with the notable exceptions of snRNA and a small percentage of mRNAs, Ψ levels in these RNA fractions will not be affected by dyskerin knockdown. Even though rRNAs constitute over 80% of total cellular RNA [35], the nascent rRNA population residing in the nucleus is expected to be substantially less. In this case, our modest reduction in Ψ levels could also be explained by the relative abundance of co-purifying tRNA and mRNA in our analyte.

Previously, functional studies of Ψ focused on the different pseudouridylation levels at specific Ψ residues in rRNA, tRNA, and mRNA [4,8,36]. While this information is invaluable to study the mechanism of Ψ regulation, mapping the changes in individual Ψ targets is time-consuming and may not reflect comprehensive Ψ regulation. With our assay, the global Ψ levels in a specific RNA pool can be quantified to serve as a quick readout of changes in Ψ levels (either in total RNA or in an isolated RNA pool) in response to different types of cellular stress including, but not limited to, heat shock, hypoxia, and modified growth conditions. The changes in global Ψ levels, either in a dose–response or in a temporal manner, can then be correlated with viability, toxicity and/or other functional endpoints. Once the consequences associated with pseudouridylation defects are observed, further studies could then be performed to determine the mechanisms, i.e., screening for the target residues of pseudouridylation that are responsible for the outcome.

With only the digestion step prior to analysis, our method provides accurate quantification of Ψ levels without any additional sample derivatization or manipulation. It has been used to measure Ψ levels in vivo and to measure changes in pseudouridylation over time. Our fast and economical HPLC-UV method can also be applied to detect and determine low levels of Ψ in different RNA fractions with small amounts of RNA input.

## Figures and Tables

**Table 1:** Retention times for major nucleosides using the HPLC-UV method.

Analyte	Retention Time (min)
Ψ	2.0
Cytosine	2.7
Uridine	3.7
7-methyl Guanosine (I.S.)	6.5
Guanosine	7.2
Adenosine	12.7

I.S.: internal standard analyte.

**Table 2:** Accuracy and precision of the HPLC-UV method.

Spiked Conc. (pmol/μL)	Intra-Day (*n* = 3)			Inter-Day (*n* = 3)		
	Observed Conc. (pmol/μL)	Precision (RSD, %)	Accuracy (RE, %)	Observed Conc. (pmol/μL)	Precision (RSD, %)	Accuracy (RE, %)
0.19	0.20 ± 0.01	5.70	104.19	0.21 ± 0.02	8.34	108.12
1.54	1.66 ± 0.01	0.50	108.07	1.66 ± 0.04	2.92	108.70
12.28	12.90 ± 0.25	1.86	107.02	12.90 ± 0.26	2.00	106.37

Conc.: concentration; RSD: relative standard deviation; RE: relative error.

**Table 3:** Average Ψ levels in different RNA pools.

Cell Line	Fraction	RNA Size (nt)	Conc. (pmol/μL)	Ψ%	RSD (%)
HEK293	Nuclear-enriched RNA	N/A	0.88	1.20%	8.84%
HEK293	Small RNA	75–121	1.43	1.94%	14.77%
Hela	18S rRNA	1868	1.30	1.77%	0.87%
Hela	28S rRNA	5025	0.72	0.98%	0.76%

## Supplementary Materials

The following are available online at www.mdpi.com/2073-4425/8/9/219/s1.
