# Supplementary material for: Quantification of Pseudouridine Levels in Cellular RNA Pools with a Modified HPLC-UV Assay
Source: Genes (Basel). 2017 Sep 5;8(9):219. doi: 10.3390/genes8090219 (PMC5615352; doi:10.3390/genes8090219)
Supplement: Supplementary file 1 [file genes-08-00219-s001.docx]

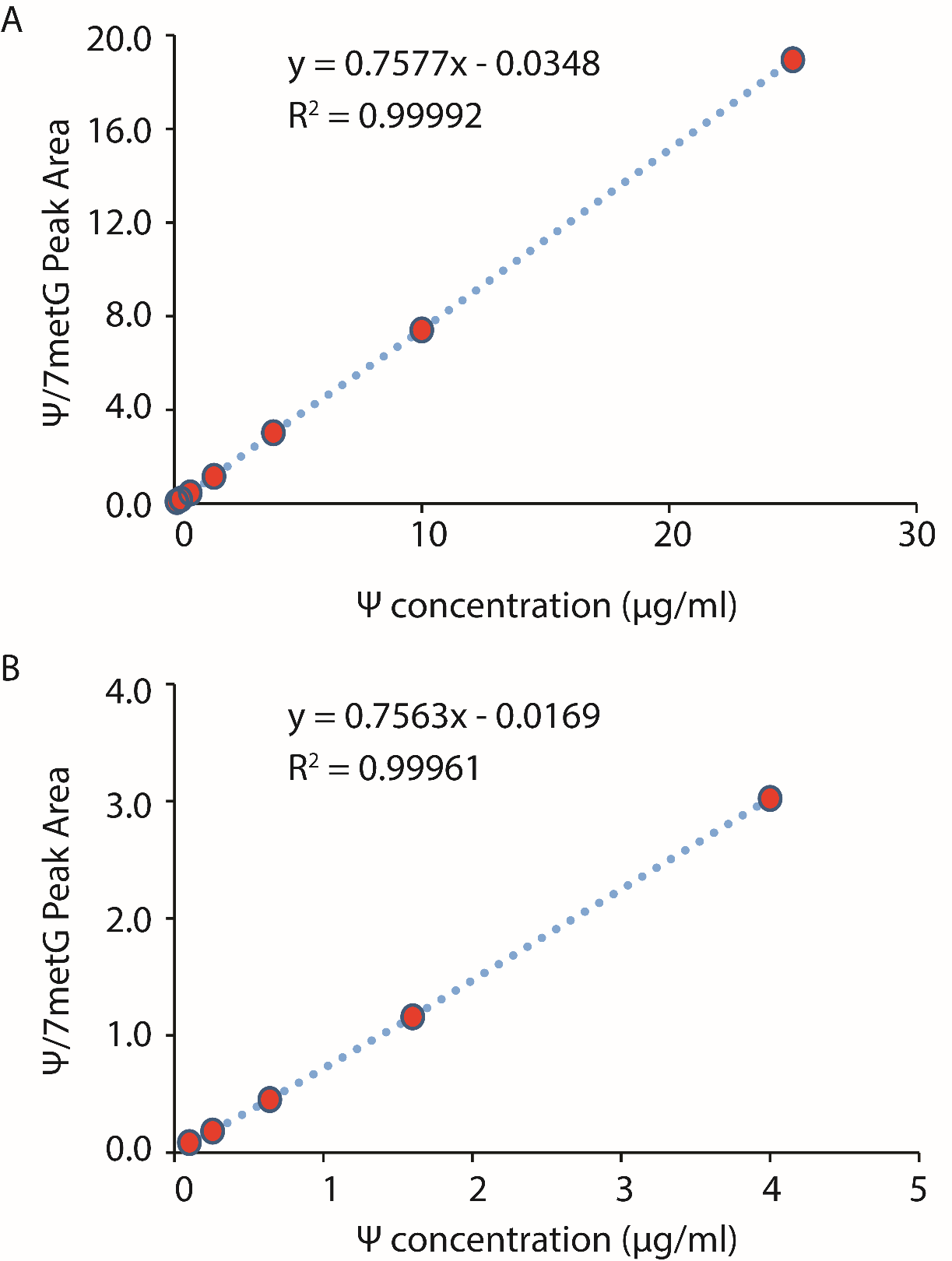


**Figure S1.** Representative calibration curves and corresponding equations as well as regression coefficients (R^2^) were shown below. (**A**) The calibration curve with all the calibration standards (n = 7) was used for the calculations in the paper. The concentrations of the working solutions were used. (**B**) The area in the lower concentration range of the calibration curve (n = 5) was amplified to show that the slope of the calibration curve was not driven by values at the high ends.
